# Relevance of spirituality and perceived social support to mental health of people with pre-existing mental health disorders during the COVID-19 pandemic: a longitudinal investigation

**DOI:** 10.1007/s00127-023-02590-1

**Published:** 2023-12-19

**Authors:** Franziska Tutzer, Timo Schurr, Beatrice Frajo-Apor, Silvia Pardeller, Barbara Plattner, Anna Schmit, Andreas Conca, Martin Fronthaler, Christian Haring, Bernhard Holzner, Markus Huber, Josef Marksteiner, Carl Miller, Verena Perwanger, Roger Pycha, Martin Schmidt, Barbara Sperner-Unterweger, Alex Hofer

**Affiliations:** 1 Division of Psychiatry I, Department of Psychiatry, Psychotherapy, Psychosomatics and Medical Psychology, Medical University Innsbruck, Anichstr. 35, 6020 Innsbruck, Austria; 2 Department of Psychiatry, General Hospital of Bolzano, Sanitary Agency of South Tyrol, Bolzano, Italy; 3 Therapy Center Bad Bachgart, Sanitary Agency of South Tyrol, Rodengo, Italy; 4 Department of Psychiatry and Psychotherapy B, State Hospital Hall in Tyrol, Hall in Tyrol, Austria; 5 Department of Psychiatry, General Hospital of Brunico, Sanitary Agency of South Tyrol, Brunico, Italy; 6 Department of Psychiatry and Psychotherapy A, State Hospital Hall in Tyrol, Hall in Tyrol, Austria; 7 Department of Psychiatry, County Hospital Kufstein, Kufstein, Austria; 8 Department of Psychiatry, General Hospital of Merano, Sanitary Agency of South Tyrol, Merano, Italy; 9 Department of Psychiatry, General Hospital of Bressanone, Sanitary Agency of South Tyrol, Bressanone, Italy; 10 Department of Psychiatry, County Hospital Lienz, Lienz, Austria; 11 Division of Psychiatry II, Department of Psychiatry, Psychotherapy, Psychosomatics and Medical Psychology, Medical University Innsbruck, Innsbruck, Austria

**Keywords:** COVID-19, Psychological distress, Spirituality, Perceived social support

## Abstract

**Background:**

The COVID-19 pandemic and related measures have negatively impacted mental health worldwide. The main objective of the present longitudinal study was to investigate mental health in people living in Tyrol (Austria) and South Tyrol (Italy) during the COVID-19 pandemic and to report the prevalence of psychological distress among individuals with versus those without pre-existing mental health disorders (MHD) in the long-term (summer 2020–winter 2022). Here, we specifically focus on the relevance of spirituality and perceived social support in this regard.

**Methods:**

161 individuals who had been diagnosed with MHD and 446 reference subjects participated in this online survey. Electronic data capture was conducted using the Computer-based Health Evaluation System and included both sociodemographic and clinical aspects as well as standardized questionnaires on psychological distress, spirituality, and the perception of social support.

**Results:**

The prevalence of psychological distress was significantly higher in individuals with MHD (36.6% vs. 12.3%) and remained unchanged among both groups over time. At baseline, the perception of social support was significantly higher in healthy control subjects, whereas the two groups were comparable in regards of the subjective relevance of faith. Reference subjects indicated significantly higher spiritual well-being in terms of the sense of meaning in life and peacefulness, which mediated in large part the between-group difference of psychological distress at follow-up. Notably, both faith and the perception of social support did not prove to be relevant in this context.

**Conclusions:**

These findings point to a consistently high prevalence of psychological distress among people suffering from MHD and underscore the prominent role of meaning in life and peacefulness as a protective factor in times of crisis. Therapeutic strategies that specifically target spirituality may have a beneficial impact on mental health.

## Introduction

The COVID-19 pandemic and related measures such as quarantine, lockdowns, and social isolation have negatively impacted mental health worldwide [1] and have been associated with increased levels of anxiety, depression, frustration, insecurity, agitation, sleep disturbances, and boredom [2, 3]. Similar outcomes have already been observed during earlier epidemics like SARS, MERS, and Ebola virus disease [4].

In line with the findings from other countries, our group has recently shown that the COVID-19 pandemic has a negative impact on the psychological condition of the residents of Tyrol (Austria) and South Tyrol (Italy). At the early stages of the pandemic, approximately 15% of the study participants out of the general population reported on psychological distress with women, singles, low-income people, as well as those who were unemployed being particularly affected [5, 6]. This prevalence was markedly higher in patients suffering from severe mental illnesses (schizophrenia spectrum disorder, bipolar disorder, major depressive disorder with psychotic features: 23.9%) and significantly higher in patients suffering from major depressive disorder without psychotic features (approximately 45%) [7]. Notably, the prevalence of clinically relevant psychological symptoms remained unchanged among each group over time and a higher degree of resilience and extraversion as well as less loneliness and boredom predicted reduced psychological distress in the short-term [7, 8]. Yet, still other coping strategies and protective factors exist to deal with psychological distress in the context of a crisis, e.g., religiosity and spirituality which are well-known coping factors with a positive effect on physical and mental health [9, 10], as well as social support [7, 11].

Religiosity and spirituality may seem very similar at first glance. Religiosity refers to a person's behavior and attitudes toward a particular religion and its values, rules, and practices [12]. Spirituality, on the other hand, can be defined as the way individuals search for meaning and purpose in life, how they relate to and connect with themselves, others, the moment, nature, or even the saints [13], and it can also be understood as an inner belief system that gives meaning and vitality to all events and to life itself [14]. Accordingly, spiritual-religious coping means that religious beliefs, attitudes, and practices are used to reduce emotional distress caused by events beyond personal control in order to give meaning to suffering and making it more bearable [15]. For example, Schuster and co-workers have shown that after the terrorist attacks of 2001 people turned to religion and spirituality to cope better with those events [16] and similar findings have been obtained after war scenarios [17]. In general, spiritual individuals find it easier to cope with loss and the grief for loved ones [18]. However, spirituality is not only a good coping mechanism to deal with stressful situations but also a common way to cope with illness and chronic diseases [19, 20]. On the other hand, it has played a subordinate role in the treatment of mental health disorders (MHD)so far, yet many patients would like spirituality to become a relevant element of their therapy concept [21].

Numerous previous studies have emphasized the meaning of social support for physical and psychological well-being [22] and accordingly, the dramatic consequences of pandemic-associated social restrictions on mental health are not surprising [23]. For example, even before the pandemic, Peirce et al. have shown that social contact is positively related to perceived social support, which, in turn, is negatively associated with depression [24]. It has to be noted, however, that the social support actually received may correlate poorly with perceived social support [25, 26] and that the relationship between received social support and mental health may be weak [26–28]. In contrast, there is a strong negative association between social support and stress perception [29] and a strong positive association between perceived social support and mental health in general [30, 31]. For example, perceiving social support during the early phases of the COVID-19 pandemic was associated with a lower risk for depression [32].

Taking into consideration a history of MHD, the main objective of the present longitudinal study was to investigate the mental health of people living in Tyrol (Austria) and South Tyrol (Italy) during the COVID-19 pandemic. These populations have similar characteristics and are comparable in many ways (socio-economic context, health system, etc.) [33], however, due to rapid dissemination of SARS-CoV-2 and an overburdened national health system, public health policy measures to contain the pandemic were much stricter in Italy. Interestingly, the prevalence of psychological distress was comparable between study participants from Tyrol and South Tyrol in the short term and as expected, we found evidence for a particular burden in people with pre-existing MHD [7]. We now report the prevalence of psychological distress among individuals with versus those without pre-existing MHD in the long-term (summer 2020–winter 2022) focusing in particular on the relevance of spirituality and perceived social support in this regard.

## Methods

### Participants

3928 residents of Tyrol and 1587 residents of South Tyrol aged 18 and above who had been diagnosed with MHD during an inpatient stay at one of the local psychiatric wards in 2019 were invited by mail to participate in a longitudinal online survey. In parallel, reference subjects from the general population were recruited through advertising in print media, email lists, flyers, and social media. They were asked to provide an email address to be reminded for follow-up, however, this was not a prerequisite to participate in the baseline survey. Reference subjects under the age of 18 years or reporting to have been diagnosed with a MHD in the past as well as those reporting on current psychopharmacological and/or psychotherapeutic treatment were excluded from further analyses.

So far, three surveys have been conducted. For organizational reasons, the collection of baseline (T0) and short-term follow-up (T1) data took place at different dates in the two countries, however, the time interval between those surveys was equal (11 weeks). In Tyrol, T0 was conducted between June 26, 2020 and September 13, 2020 (South Tyrol: September 7, 2020–November 22, 2020) and T1 between November 30, 2020 and January 24, 2021 (South Tyrol: February 8, 2021–April 4, 2021). In both countries, the long-term follow-up (T2) was conducted between January 10, 2022 and February 21, 2022. At the end of each survey, participants received a downloadable information sheet on professional support numbers and addresses. The results of short-term follow-up have been reported previously [7, 8, 34]. Here, we focus on the findings of long-term follow-up.

### Procedures

The study was approved by the Ethics Committees of the Medical University Innsbruck, Austria (Approval No. 1147/2020) and of the Sanitary Agency of South Tyrol, Italy (Approval No. 83/2020) and all participants provided informed consent online. Electronic data capture was conducted using the Computer-based Health Evaluation System (CHES) [35] and included both sociodemographic and clinical aspects as well as standardized questionnaires.

Psychological distress was assessed using the 53-item Brief Symptom Checklist (BSCL) [36]. This is a Likert-type scale whose items are rated from 0 (not at all/no distress) to 4 (extremely/very strong distress). Nine symptom groups of mental health problems are examined: anger-hostility, anxiety, depression, paranoid ideation, phobic anxiety, psychoticism, somatization, interpersonal sensitivity, and obsessive-compulsiveness. The Global Severity Index (GSI) used in the current study was calculated using the sum of the nine symptom dimensions plus four additional items not included in any of the dimension scores divided by the total number of answered items. Additionally, BSCL raw scores and GSI composite scores were transformed into sex- and age-specific normative *T*-scores (average: 50 ± 10) using a standardization reference table. A *T*-score ≥ 63 was defined to indicate clinically relevant psychological symptoms.

Spirituality was assessed with the Functional Assessment of Chronic Illness Therapy—Spiritual Well-Being Non-Illness Version (FACIT-Sp Non-Illness) [37]. The FACIT-Sp Non-Illness is a collection of health-related quality of life questionnaires that includes eight questions relating to the sense of meaning in life and peacefulness (FACIT-Sp MP) and four questions relating to the sense of strength and comfort from one’s faith (FACIT-Sp F), respectively. Of note, the FACIT-Sp is scored without referral to religious beliefs or practice. Each item is scored between 0 and 4 with higher scores indicating higher spiritual well-being.

Perceived social support was assessed with the Multidimensional Scale of perceived social support (MSPSS) [38], a 12-item self-report scale that measures perceptions and adequacy of social support from three sources: family, friends, and significant others. Each subscale includes four items: practical help, emotional support, availability to discuss problems, and help with decision making. The total score reflects the total degree of social support that individuals receive. Items were scored on a 5-point Likert scale ranging from 1 = “strongly disagree” to 5 = “strongly agree”. Scores > 50% indicate high perceived support.

### Statistical analysis

Initially, metric variables were checked for deviations from normal distribution by visual inspection and investigating skewness and kurtosis. Cook’s distance, Mahalanobis distance, and leverage values were calculated to identify possible (multivariate) outliers. The statistical significance level was set to alpha = 5%. Depending on variable type, Chi^2^ test (dichotomous, categorical) and Mann–Whitney-*U* test (non-normally distributed metric) was applied for comparisons between patients and the reference group regarding sociodemographic data and psychological variables. For longitudinal comparisons between baseline and follow-up within the patient and the reference group the McNemar test was used for dichotomous variables and the Wilcoxon signed ranks test for (non)normally distributed metric variables. Possible associations between age, years of education, mediator, and dependent variables within the patient and the reference group at baseline and follow-up were analyzed by Spearman rank correlations. Fisher’s *z*-transformed correlation coefficients within the patient and the reference group were calculated and compared. Effect sizes can be interpreted as follows: *r*, *V* = 0.10–0.29; *d* = 0.2–0.4 small; *r*, *V* = 0.30–0.49; *d* = 0.5–0.7 medium, and *r*, *V* ≥ 0.50; *d* ≥ 0.8 high [39]. An odds ratio of 1.0 indicates that there is no difference between the patient and the reference group. An odds ratio > 1.0 indicates increased odds for the patient group, and an odds ratio < 1.0 indicates decreased odds for the patient group.

A logistic regression was conducted to find possible explanations for the high number of dropouts between baseline and follow-up measurement. This analysis included the following independent baseline variables: age, sex, residence, relationship status, years of education, physical health problems, spirituality, perceived social support, and psychological distress.

PROCESS v4.0 [40] was used to carry out the mediation analyses. This macro provides path coefficients for the direct, indirect, and total effects by means of ordinary least square regressions. Reported heteroscedasticity-consistent standard errors (HC3) [41] and the determined 95% confidence intervals are based on 10.000 percentile bootstrapped samples. When the confidence interval did not include zero, indirect effects were considered statistically significant. Interactions between independent and mediator variables were deemed statistically significant when *p* was < 0.05. Subjects (patients/reference group) were included as independent grouping variable. Spirituality (FACIT-Sp MP, FACIT-Sp F) and perceived social support (MSPSS) measured at baseline were assigned as mediator variables, whereas psychological distress (GSI) at follow-up was used as the dependent variable. Since patients and reference subjects differed significantly regarding the baseline variables sex, age, years of education, relationship status, and physical health problems, these variables were included in an initial mediation model to probe their explanatory contribution. Furthermore, residence and psychological distress at baseline were included during model building procedure. Yet, due to the strong association with the grouping factor, relationship status and physical health problems were not included as covariates in the final mediation analysis. Otherwise, there would have been the risk of overfitting the model, possibly leading to biased estimates.

## Results

Out of 5517 patients suffering from MHD who had been invited for study participation 443 took part in the baseline survey. 185 completed both baseline and long-term follow-up surveys. However, patients diagnosed with behavioral syndromes associated with physiological disturbances and physical factors (ICD-10: F5x.xx) as well as those diagnosed with disorders of adult personality and behavior (ICD-10: F6x.xx) could not be considered in the analyses due to uneven distribution of psychological distress (GSI) values. This was confirmed by significant (*p* < 0.001) Kruskal–Wallis test and Bonferroni corrected pairwise comparison results. This resulted in a patient sample reduction of 14.8% (*n* = 28). Additionally, 1642 reference subjects fulfilling the inclusion criteria participated in the baseline survey, of whom 446 completed both baseline and follow-up surveys and were included in the analyses of the current report. Results of the logistic regression analysis revealed that age (OR = 0.976, 95% CI [0.967–0.985], *p* < 0.001), meaning and peacefulness (OR = 0.961, 95% CI [0.932–0.990], *p* = 0.009), and faith (OR = 1.043, 95% CI [1.013–1.074], *p* = 0.005) at baseline were associated with dropout at follow-up assessment.

Table 1 shows the baseline characteristics of study participants. Patients were older, less educated, and more often single. In both groups, the majority of participants were female. Table 2 depicts disease- and treatment-related characteristics of patients at baseline and follow-up. At follow-up, 62.7% of patients were receiving treatment for their MHD, which constitutes a significant decrease from baseline (72.5%).

**Table 1:** Baseline characteristics of patients and reference subjects

Variable	Patients (*n* = 161)	Reference subjects (*n* = 446)	Statistics	*p*-value
	Mean ± SD or N (%)	Mean ± SD or *N* (%)		
Sex				
Male	77/161 (47.8%)	133/446 (29.8%)	*χ*^2^ = 16.95	< 0.001
Female	84/161 (52.2%)	313/446 (70.2%)		
Age (years)	49.0 ± 13.4 (19–82)	45.0 ± 13.7 (18–96)	|*Z*| 3.53	< 0.001
Education (years)	13.4 ± 4.3	15.7 ± 3.7	|*Z*| 6.78	< 0.001
Residence				
Tyrol (Austria)	113/161 (70.2%)	275/446 (61.7%)	*χ*^2^ = 3.73	0.053
South Tyrol (Italy)	48/161 (29.8%)	171/446 (38.3%)		
Relationship				
Single	58/161 (36.0%)	89/446 (20.0%)	*χ*^2^ = 17.78	< 0.001
Fixed partnership	100/161 (62.1%)	357/446 (80.0%)		
Severe physical health problems (e.g., diabetes, cancer, etc.)	26/161 (16.1%)	28/446 (6.3%)	*χ*^2^ = 14.22	< 0.001
Average years since initial diagnosis of MHD (base 2020)	11.6 ± 12.1 (median 6.0)			
Average years since first in-patient treatment due to psychiatric disorder (base 2020)	8.0 ± 10.6 (median 2.0)			
Number of patients with ICD-10 F0x.x as primary diagnosis	2/161 (1.2%)			
Number of patients with ICD-10 F1x.x as primary diagnosis	33/161 (20.5%)			
Number of patients with ICD-10 F2x.x as primary diagnosis	16/161 (9.9%)			
Number of patients with ICD-10 F3x.x as primary diagnosis	74/161 (46.0%)			
Number of patients with ICD-10 F4x.x as primary diagnosis	36/161 (22.4%)			

Always one degree of freedom unless specified otherwise

MHD, mental health disorders; SD, standard deviation

**Table 2:** Disease-/ and treatment-related characteristics of patients at baseline and long-term follow-up

Variable	Baseline	Follow-up
Current treatment due to psychiatric disorder	116/161 (72.5%)	101/161 (62.7%)↓↓
Psychological/psychotherapeutic treatment	72/116 (62.0%)	61/101 (60.4%)
Psychiatric treatment (outside a hospital)	72/116 (62.0%)	67/101 (66.3%)
Psychiatric treatment (outpatient unit of a hospital)	41/116 (35.3%)	31/101 (30.7%)
General practitioner	27/116 (23.3%)	23/101 (22.8%)
Care facility (work)	10/116 (8.6%)	9/101 (8.9%)
Care facility (living)	2/116 (1.7%)	4/101 (4.0%)
Experienced any change in the course of the psychiatric illness since the onset of the COVID-19 pandemic	–	69/161 (42.9%)
Use of outpatient and complementary mental health services since the beginning of the pandemic has been…	–	43/161 (26.7%)
Increased	–	23/43 (53.5%)
Decreased	–	20/43 (46.5%)
Inpatient treatment for the psychiatric condition since the onset of the COVID-19 pandemic	–	37/161 (23.0%)
Perceived connection between the pandemic and the deterioration in the state of health that led to hospitalization	–	11/37 (29.7%)
Change of medication since the start of the COVID-19 pandemic	–	59/161 (36.6%)

↓↓ = statistically significant (*p* < 0.01) decrease between baseline and follow-up according to McNemar-test

Table 3 provides an overview of COVID-19-related characteristics. 28.3% of the patients and 17.4% of the reference subjects had suffered from COVID-19 up to follow-up. A total of 6 subjects required hospitalization and one required ICU treatment. Among both groups, confinement and negative press were the most distressing factors. In the reference group 32.1% were burdened by spatial separation from family and/or partner and 18.6% by home schooling, while in the patients group restricted access to retail and gastronomy (28.6% each) were the most stressful factors following isolation and negative press. However, in the subjective rating scale from 0 to 10 questioning how much psychological stress the COVID-19 crisis had caused, no between-group differences could be detected (median 5.1 in both groups).

**Table 3:** COVID-19 related characteristics of patients and reference subjects measured at long-term follow-up

Variable	Patients (*n* = 161)	Reference subjects (*n* = 446)	Statistics		*p*-value
	Mean ± SD or *N* (%)	Mean ± SD or *N* (%)			
Diagnosed with COVID-19 infection	28/161 (17.4%)	126/446 (28.3%)	OR = 0.53	*χ*^2^ = 7.37	0.007
No/barely symptoms	5/161 (17.9%)	17/446 (13.5%)	*V* = 0.20	*χ*^2^ = 6.13; *df* = 4	0.189
Minor symptoms/treatment at home	10/161 (35.7%)	53/446 (42.1%)			
Symptoms with fever/treatment at home	10/161 (35.7%)	52/446 (41.3%)			
Severe symptoms/treatment at the hospital	2/161 (7.1%)	4/446 (3.2%)			
Serious symptoms/treatment at the ICU	1/161 (3.6%)	0/446 (0.0%)			
COVID-19 vaccinated	148/161 (91.9%)	413/446 (92.6%)	OR = 0.91	*χ*^2^ = 0.08	0.781
What has been most stressful to you since the outbreak of the COVID-19 pandemic?					
Home-Office	10/161 (6.2%)	40/446 (9.0%)	OR = 0.67	*χ*^2^ = 1.19	0.275
Home-Schooling	15/161 (9.3%)	83/446 (18.6%)	OR = 0.45	*χ*^2^ = 7.55	0.006
Confinement	86/161 (53.4%)	243/446 (54.5%)	OR = 0.96	*χ*^2^ = 0.05	0.816
Restricted access to retail	46/161 (28.6%)	64/446 (14.3%)	OR = 2.39	*χ*^2^ = 16.13	< 0.001
Restricted access to gastronomy	46/161 (28.6%)	139/446 (31.2%)	OR = 0.88	*χ*^2^ = 0.38	0.540
Fear of falling ill	45/161 (28.0%)	96/446 (21.5%)	OR = 1.41	*χ*^2^ = 2.74	0.098
Negative press	90/161 (55.9%)	234/446 (52.5%)	OR = 1.15	*χ*^2^ = 0.56	0.454
Spatial separation from family and/or partner	33/161 (20.5%)	143/446 (32.1%)	OR = 0.55	*χ*^2^ = 7.69	0.006
On a scale of 1 to 10, how much psychological stress did the COVID-19 crisis cause you? (1 = not at all; 10 = maximum stress)	5.00 ± 2.62 (median 5.1)	4.92 ± 2.46 (median 5.1)	*d* = 0.02	|*Z*| 0.30	0.767

Always one degree of freedom unless specified otherwise

ICU, intensive care unit; SD, standard deviation

Both at baseline (36.6% vs. 12.3%) and at follow-up (37.9% vs. 12.3%) patients were significantly more likely to be psychologically distressed than the reference subjects. In contrast, at baseline, the latter achieved significantly higher scores in the FACIT-Sp MP (25.1 ± 4.93 vs. 19.4 ± 7.40) and in the MSPSS (4.37 ± 0.61 vs. 3.86 ± 0.82), whereas the two groups were comparable in regards of FACIT-Sp F scores. Details are depicted in (Table 4)

**Table 4:** Variable characteristics of psychological distress, spirituality, and perceived social support at baseline and long-term follow-up

Variable		Patients (*n* = 161)	Reference subjects (*n* = 446)	Statistics		*p*-value
Psychological distress (BSCL)		*T* value ≥ 63% (N)	*T* value ≥ 63% (N)			
Anger-hostility	B	22.4% (36/161)	13.9% (62/446)	OR = 1.78	*χ*^2^ = 6.25	0.012
	*FU*	*13.0% (21/161)↓*	*11.4% (51/446)*	OR = 1.16	*χ*^2^ = 0.29	0.588
Anxiety	B	39.1% (63/161)	14.3% (64/446)	OR = 3.84	*χ*^2^ = 43.91	< 0.001
	*FU*	*36.0% (58/161)*	*14.1% (63/446)*	OR = 3.42	*χ*^2^ = 35.55	< 0.001
Depression	B	29.2% (47/161)	9.0% (40/446)	OR = 4.19	*χ*^2^ = 39.41	< 0.001
	*FU*	*27.3% (44/161)*	*8.3% (37/446)*	OR = 4.16	*χ*^2^ = 37.06	< 0.001
Paranoid ideation	B	23.6% (38/161)	9.0% (40/446)	OR = 3.14	*χ*^2^ = 22.62	< 0.001
	*FU*	*25.5% (41/161)*	*9.9% (44/446)*	OR = 3.12	*χ*^2^ = 23.91	< 0.001
Phobic anxiety	B	52.8% (85/161)	40.6% (181/446)	OR = 1.64	*χ*^2^ = 7.17	0.007
	*FU*	*46.6% (75/161)*	*28.0% (125/446)↓↓*	OR = 2.24	*χ*^2^ = 18.44	< 0.001
Psychoticism	B	41.0% (66/161)	12.6% (56/446)	OR = 4.84	*χ*^2^ = 59.57	< 0.001
	*FU*	*33.5% (54/161)*	*11.9% (53/446)*	OR = 3.74	*χ*^2^ = 38.21	< 0.001
Somatization	B	19.3% (31/161)	8.1% (36/446)	OR = 2.72	*χ*^2^ = 15.07	< 0.001
	*FU*	*21.1% (34/161)*	*9.2% (41/446)*	OR = 2.65	*χ*^2^ = 15.34	< 0.001
Interpersonal sensitivity	B	28.0% (45/161)	8.7% (39/446)	OR = 4.05	*χ*^2^ = 36.60	< 0.001
	*FU*	*28.0% (45/161)*	*11.4% (51/446)*	OR = 3.01	*χ*^2^ = 24.23	< 0.001
Obsessive-compulsiveness	B	34.2% (55/161)	10.5% (47/446)	OR = 4.41	*χ*^2^ = 47.22	< 0.001
	*FU*	*29.8% (48/161)*	*15.9% (71/446)↑*	OR = 2.24	*χ*^2^ = 14.49	< 0.001
Global Severity Index	B	36.6% (59/161)	12.3% (55/446)	OR = 4.11	*χ*^2^ = 45.85	< 0.001
	*FU*	*37.9% (61/161)*	*12.3% (55/446)*	OR = 4.34	*χ*^2^ = 49.98	< 0.001
		Mean ± SD	Mean ± SD			
		Median (range)	Median (range)			
Global Severity Index	B	0.83 ± 0.71	0.42 ± 0.42	*d* = 0.58	|*Z*| 6.99	< 0.001
		0.68 (0–2.87)	0.28 (0–2.11)			
	*FU*	*0.81 ± 0.66*	*0.45 ± 0.43*^*a*^	*d* = 0.58	|*Z*| 6.78	< 0.001
		0.66 (0–2.96)	0.32 (0–2.77)			
Spirituality (FACIT-SP)		Mean ± SD	Mean ± SD			
		Median (range)	Median (range)			
Meaning/peacefulness	B	19.4 ± 7.40	25.1 ± 4.93	*d* = 0.80	|*Z*| 9.13	< 0.001
		20.0 (0–32)	26.0 (4–32)			
Faith	B	6.28 ± 4.66	6.45 ± 4.09	*d* = 0.10	|*Z*| 1.19	0.234
		6.00 (0–16)	5.50 (0–16)			
Perceived social support		Mean ± SD	Mean ± SD			
		Median (range)	Median (range)			
MSPSS	B	3.86 ± 0.82	4.37 ± 0.61	*d* = 0.58	|*Z*| 6.99	< 0.001
		4.00 (1.75–5)	4.50 (1.75–5)			
		% (N)	% (N)			
	Low (range 12–25)	3.7% (6/161)	0.4% (2/446)	*V* = 0.28	*χ*^2^ = 46.92; *df* = 2	< 0.001
	Medium (range 26–43)	30.4% (49/161)	10.5% (47/446)			
	High (range 44–60)	65.8% (106/161)	89.0% (397/446)			

BSCL, Brief Symptom Checklist; FACIT-SP, Functional Assessment of Chronic Illness Therapy—Spiritual Well-Being; Non-Illness Version; MSPSS, Multidimensional Scale of Perceived Social Support; B, Baseline; FU, Follow-Up; SD, standard deviation

↓ = statistically significant (*p* < 0.05) decrease between baseline and follow-up according to McNemar-test

↑ = statistically significant (*p* < 0.05) increase between baseline and follow-up according to McNemar-test

↓↓ = statistically significant (*p* < 0.001) decrease between baseline and follow-up according to McNemar-test

^a^Statistically significantly (*p* < 0.05) higher at follow-up compared to baseline according to Wilcoxon Signed Ranks Test (two-tailed)

Note: Always one degree of freedom unless specified otherwise

.

Table 5 shows the correlations between age, years of education, psychological distress (GSI), spirituality (FACIT-Sp MP, FACIT-SP F), and perceived social support (MSPSS). Among both groups, simultaneous assessments at baseline revealed a strong negative correlation between GSI and FACIT-Sp MP scores and a moderate negative correlation between GSI and MSPSS scores. In addition, the GSI score was moderately negatively correlated with age and the FACIT-Sp F score in patients only.

**Table 5:** Spearman rank correlations of variables for mediation analysis within the patient and reference group

Group	Measure	Age	Years of education	Meaning/peace	Faith	Social support	Psychological distress (baseline)
Patients (*n* = 161)	Years of education	− 0.172*					
	Meaning/peacefulness (baseline)	0.225**	− 0.055				
	Faith (baseline)	0.135	− 0.092	0.601***			
	Perceived social support (baseline)	− 0.046	− 0.046	0.410***	0.262***		
	Psychological distress (baseline)	− 0.336***	0.119	− 0.746***	− 0.364***	− 0.342***	
	Psychological distress (follow-up)	− 0.241**	0.063	− 0.574***	− 0.282***	− 0.315***	0.678***
Reference subjects (*n* = 446)	Years of education	− 0.146**					
	Meaning/peacefulness (baseline)	0.062	− 0.032				
	Faith (baseline)	0.042	− 0.107*	0.256***			
	Perceived social support (baseline)	− 0.223***	0.032	0.453***	0.203***		
	Psychological distress (baseline)	− 0.080	− 0.032	− 0.561***	− 0.076	− 0.344***	
	Psychological distress (follow-up)	− 0.084	− 0.049	− 0.532***	− 0.084	− 0.271***	0.711***

Fisher’s *z* transformed testing of correlation coefficients differed statistically significantly between both groups (patients vs. reference subjects) regarding the following comparisons

Age versus meaning/peacefulness (*z* = 1.80, *p* = 0.036);

Age versus perceived social support (*z* = 1.95, *p* = 0.026);

Age versus psychological distress [baseline] (*z* = − 2.91, *p* = 0.002);

Age versus psychological distress [follow-up] (*z* = − 1.77, *p* = 0.041);

Psychological distress [baseline] versus meaning/peacefulness (*z* = − 3.56, *p* < 0.001);

Psychological distress [baseline] versus faith (*z* = − 3.30, *p* < 0.001);

Faith versus meaning/peacefulness (*z* = − 4.67 p < 0.001);

Faith versus psychological distress [baseline] (*z* = − 2.22, *p* = 0.013)

*p < 0.05; **p < 0.01; ***p < 0.001

In patients, significant negative correlations were detected between age (weak correlation) as well as baseline FACIT-Sp MP (strong correlation), MSPSS (moderate correlation), and FACIT-SP F values and GSI at follow-up. In reference subjects, in turn, merely baseline FACIT-Sp MP (strong correlation) and MSPSS (weak correlation) values correlated significantly negatively with GSI at follow-up. Further details are depicted in Table 4.

### Results of mediation analysis

The analysis for (multivariate) outliers using Mahalanobis distance, Cooks distance, and leverage values indicated that the previously set limits were not exceeded. Thus, no outliers were detected. The analysis regarding possible interaction effects between the independent grouping variable and the mediators did not yield statistically significant results.

**Fig. 1:**
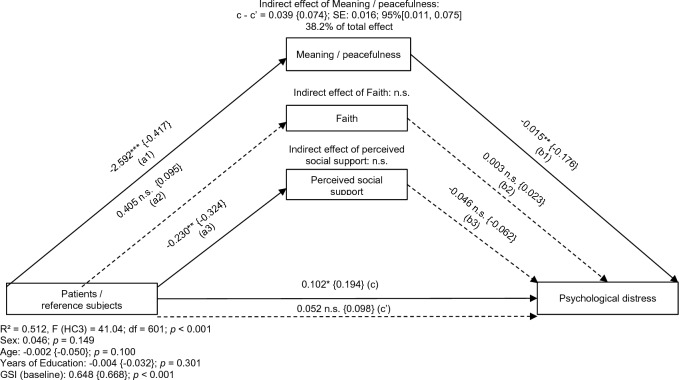
Mediation model (*n*=607) with the effect of patients and reference subjects on psychological distress (GSI) at follow-up mediated by the sense of meaning in life and peacefulness (FACIT-Sp MP), the sense of strength and comfort from one’s faith (FACIT-Sp F), and perceived social support (MSPSS) measured at baseline. *Abbreviations*: GSI, Global Severity Index; n.s., not significant; SE, standard error. *Note*: Reference subjects are coded with (0), patients are coded with (1). Values in curved brackets represent completely standardized coefficients (β) for metric and partially standardized coefficients for dichotomous variables. Coefficient of determination (*R*²), coefficients and *p*-values of covariates included in the model (sex, age, years of education, and GSI at baseline) are depicted for the total effect model. Heteroscedasticity-consistent standard errors (HC3) and 95% confidence intervals are based on 10.000 percentile bootstrapped samples. Solid lines indicate statistically significant connections. *p < 0.05; **p < 0.01; ***p < 0.001

As can be seen in Fig. 1, patients achieved significantly lower scores on the FACIT-Sp MP compared to the reference group (*a*1 = − 2.592), and participants achieving higher FACIT-Sp MP scores at baseline described lower levels of psychological distress at follow-up (*b*1 = − 0.015). Compared to the reference subjects, patients were 0.039 units higher on the GSI considering the result of the effect of group differences on meaning and peacefulness, which, in turn, supposedly affected psychological distress.

When additionally including relationship status (38.6%), residence (40.9%), and physical health (37.3%; combined [40.8%]) as covariates, in the final model (Fig. 1) 38.2% of the total effect of the grouping variable on psychological distress could be accounted for by meaning and peace.

Concerning FACIT-Sp F, there was neither a statistically significant difference in baseline faith scores between patients and reference subjects (*a*2) nor was there a significant association between FACIT-Sp F and GSI scores at follow-up (*b*2).

Compared to patients, reference subjects indicated a significantly higher perception of social support at baseline (*a*3 = − 0.230), however, there were no statistically significant associations between the perception of social support (MSPSS) and psychological distress (GSI) at follow-up (*b*3).

On average, patients experienced statistically significantly more psychological distress at follow-up when the sense of meaning in life and peacefulness (FACIT-Sp MP), the sense of strength and comfort from one’s faith (FACIT-Sp F), and perceived social support (MSPSS) were considered (*c* = 0.102). However, when the mediator variables were not taken into account, this connection could not be shown (non-significance of *c*′).

## Discussion

Our results show that patients suffering from MHD are significantly more burdened during the COVID-19 pandemic than healthy control subjects, both in the short—[8, 34] and in the long-term. At baseline, high perceived social support was significantly less frequently detected in patients, which corroborates the findings of previous studies [7, 34, 42]. On the other hand, the two groups were comparable concerning the subjective relevance of faith, while reference subjects indicated significantly higher spiritual well-being in terms of the sense of meaning in life and peacefulness, which mediated in large part the between-group difference of psychological distress at follow-up. Notably, both faith and the perception of social support did not prove to be relevant in this context.

Among both groups, the baseline assessment of psychological distress revealed a strong negative association with simultaneously assessed spiritual well-being in terms of meaning in life and peacefulness and a moderate negative association with perceived social support. This is consistent with the results of previous studies in both MHD patients and healthy control subjects [43–46]. In patients, a further moderate negative correlation was detected between psychological distress and age, which suggests that young MHD patients may represent a particularly vulnerable group in this regard and corroborates the findings of a number of previous studies showing that the young population is particularly burdened by the COVID-19 pandemic [47–49]. In addition, we also found a moderate negative correlation between patients’ psychological distress and their sense of strength and comfort from faith. It is generally well known that religiosity and spirituality may help people to cope with acute or chronic illness [18–20] and we, therefore, hypothesize that study participants with pre-existing MHD may have relied more heavily upon their faith and may have drawn strength from it even before the outbreak of the pandemic compared to the reference group. However, this issue cannot be addressed by our data. The fact that faith played a subordinate role in the reference group consisting of members of the general population is not surprising since religious affiliation has declined by 34% since 1951 in Austria [50] and is also decreasing in the Italian population [51].

Notably, the just mentioned significant associations persisted over the course of the pandemic, i.e., baseline FACIT-Sp MP, MSPSS, and FACIT-SP F values (patients only) were negatively associated with psychological distress (GSI) at follow-up. This suggests that psychological distress may not only have been caused by social isolation as a result of the pandemic, but by the pandemic itself and associated fears, e.g., about the future or one's own health or that of relatives [52]. Although life had largely normalized and quarantine conditions had been lifted, burden remained high over time, especially among MHD patients. In fact, the prevalence of psychological distress was three times higher in individuals with MHD compared to the reference group and remained unchanged among both groups over time. This difference was mediated in large part by spiritual well-being in terms of the sense of meaning in life and peacefulness and corroborates the findings of Lucchetti and coworkers who examined the association between religious and spiritual beliefs and the consequences of social isolation during the COVID-19 pandemic. In that study, spiritual participants showed lower levels of fear, anxiety, and sadness and higher levels of hope. Overall, higher spirituality was associated with better health outcomes [53]. Similarly, religious coping mechanisms such as reading the Bible or the Quran [54] and the perception of social support [55–58] also proved to be effective strategies to reduce stress, anxiety, and negative feelings in the context of the pandemic. However, the differences between MHD and control subjects in regards of psychological distress were not attributable to the mediating effect of spirituality in the sense of strength and comfort from faith or perceived social support in our survey. This suggests that independently of specific religious aspects, spirituality relating to the sense of meaning in life and peacefulness contributes to coping with crises such as the COVID-19 pandemic. In line with this consideration, spiritual interventions may improve both mental and physical health [59–63]. An experimental study has, for example, shown that spiritual interventions promote positive coping as well as the mental health of adult refugees [64]. However, there is still a lack of evidence-based spiritual interventions and further research on how they affect psychological distress caused by the COVID-19 pandemic is urgently needed.

Our study also has some limitations. Participation in the online study was voluntary and all results are based on self-report and may thus be biased. Furthermore, the reference group had to self-report of never having suffered from a mental illness. This clearly limits generalizability of our results to the entire population of Tyrol and South Tyrol. Another limiting factor is the lack of pre-pandemic data which would have been suitable as a reference point for comparison. In addition, the decreasing response rate from baseline to follow-up was associated with age, meaning and peacefulness, and faith. Younger participants and those with lower baseline scores regarding meaning and peacefulness and higher scores regarding faith were more likely to drop out. Therefore, the variability or the range of age and FACIT scores may be limited in our sample, possibly leading to limitations regarding the interpretability of the results. In summary, our results show that the prevalence of psychological distress during the pandemic was consistently higher among MHD patients compared to a healthy control group and that differences in spiritual well-being in terms of the sense of meaning in life and peacefulness were of major relevance in this regard. It remains to be seen whether the strengthening of spiritual well-being in the context of therapeutic inventions decreases psychological distress in MHD patients and ultimately leads to improved outcomes.

## Data Availability

The data that support the findings of this study are available on reasonable request from the corresponding author. The data are not publicly available due to ethical concerns, as data contains information that could compromise the privacy of research participants.
